# Perceived Shared Condemnation Intensifies Punitive Moral Emotions

**DOI:** 10.1038/s41598-017-07916-z

**Published:** 2017-08-04

**Authors:** Naoki Konishi, Tomoko Oe, Hiroshi Shimizu, Kanako Tanaka, Yohsuke Ohtsubo

**Affiliations:** 1 Department of Psychology, Graduate School of Humanities, Kobe University, Kobe, Japan; 2 Department of Psychology, Faculty of Liberal Arts, Teikyo University, Hachioji, Japan; 3 School of Sociology, Kwansei Gakuin University, Nishinomiya, Japan; 4 Department of Psychology, Faculty of Letters, Kobe University, Kobe, Japan

## Abstract

Punishment facilitates large-scale cooperation among humans, but how punishers, who incur an extra cost of punishment, can successfully compete with non-punishers, who free-ride on the punisher’s policing, poses an evolutionary puzzle. One answer is by coordinating punishment to minimise its cost. Notice, however, that in order to effectively coordinate their punishment, potential punishers must know in advance whether others would also be willing to punish a particular norm violator. Such knowledge might hinder coordination by tempting potential punishers to free-ride on other punishers. Previous research suggests that moral emotions, such as moral outrage and moral disgust, serve as a commitment device and drive people to carry out the costly act of punishment. Accordingly, we tested whether the perception of socially shared condemnation (i.e., knowledge that others also condemn a particular violator) would amplify moral outrage and moral disgust, and diminish empathy for the violator. Study 1 (scenario-based study) revealed that perceived shared condemnation was correlated positively with moral outrage and moral disgust, and negatively with empathy. Study 2 experimentally demonstrated that information indicating that others also condemn a particular norm violation amplified moral outrage. Lastly, Study 3 (autobiographical recall study) confirmed the external validity of the finding.

## Introduction

Moral sentiments and sanctions are included in Donald Brown’s list of human universals^1, 2^. These two features are conceived as important ingredients of large-scale cooperation^3, 4^. According to strong reciprocity theory, humans are not only cooperative, but also inclined to punish norm violators. Experimental studies have revealed that people punish violators of various norms, such as norms of cooperation, fairness, and honesty, even when it means incurring costs^5–11^. Costly punishment in economic games has been observed in many small-scale societies, such as hunter-gatherer societies^12–14^ and among young children^15, 16^. However, critics argue that real-life costly third-party punishments are rarely observed in small-scale societies^17, 18^. Theoretically, it is difficult to explain how punishers, who incur extra costs of punishment, can outcompete second-order free-riders, who do not punish norm violators^19–21^. In fact, field research indicates that although punishment is essential to maintain cooperation, people tend to minimise its cost by coordinating punishment against norm violators^22, 23^. Theoretically, by making his/her punishment decision contingent on other group members’ willingness to punish a particular norm violator, each punisher can avoid incurring too much punishment cost. Coordinated punishment, in effect, nullifies the fitness difference between punishers and non-punishers (i.e., second-order free-riders). A recent theoretical model shows that coordinated punishment is a viable evolutionary explanation for large-scale cooperation among humans^24, 25^.

Theoretical and empirical evidence of coordinated punishment has accumulated in the recent strong reciprocity literature^22–26^. Nevertheless, the psychological underpinnings of coordinated punishment have not been systematically investigated. To effectively coordinate their punitive behaviours, community members must solve the so-called coordination problem^27^, where each community member must make his/her punishment decision (i.e., whether to punish an apparent wrongdoer) contingent on other community members’ punishment decisions^28^; otherwise, one runs the risk of being a lone punisher, who may be perceived as a less likeable person^29–31^. The evolutionary model of coordinated punishment also predicts the evolution of conditional punishers, who punish norm violators only when a sufficient number of other punishers are present^24, 25^. Conformity may facilitate such coordination^20^. Social psychological research has revealed that people have two primary motivations for conforming to the group: to be liked and to be accurate^32, 33^. The presence of these motivations implies that people are consciously aware of the majority opinion. However, in the context of punishment, conscious awareness that the most community members are willing to punish a norm violator might be a double-edged sword: on the one hand, it fosters coordination, and on the other hand, it could worsen the second-order free-rider problem by tempting potential punishers to free-ride on other punishers.

The second-order free-rider problem may be resolved by moral emotions that counteract short-term cost-benefit calculations^34, 35^. In fact, it has been shown that moral emotions, such as moral outrage (or indignation) and moral disgust, are elicited when someone commits a norm violation^36–38^, and that these emotions motivate people to punish the norm violator even when it is costly and, thus, against their self-interest^11, 39, 40^. As such, moral emotions serve as a proximate cause of costly punishment. Therefore, coordinated punishment is facilitated if moral emotions are tuned to others’ punitive intentions. In particular, if each community member is likely to be outraged at a particular norm violation when others are also outraged, each member’s personal punishment decision is necessarily congruent with others’ punishment decisions. Accordingly, it was hypothesized that the intensity of moral emotions increases/decreases as the expectation of socially shared condemnation increases/decreases.

In addition to moral outrage and moral disgust, we examined empathy for the norm violator because several lines of research implicate empathy for norm violators as a determinant of punishment. First, having empathy for a specific criminal makes third parties’ attitudes toward criminals as a group more lenient^41^. Second, after being treated in an unfair manner, victims’ (especially male victims’) empathy for the unfair person tends to diminish, and diminished empathy predicts pleasure in seeing the unfair person suffer^42^. Finally, in a recent third-party punishment experiment, individuals low in trait empathy were more inclined to punish an unfair player^43, 44^. Based on these findings, we conjectured that diminished empathy would reduce one’s hesitation to witness the norm violator’s suffering and that this would facilitate punishment. Thus, we investigated whether empathy for the violator would be also influenced by perceived shared condemnation. In the following section, for the sake of brevity, we use the term ‘moral emotions’ rather broadly, referring not only to moral outrage and moral disgust, but also to ‘diminished’ empathy for the violator.

We conducted three studies to investigate whether perceived shared condemnation would modulate moral emotions, and they, in turn, would increase punitive motivations. Study 1, which involved 30 hypothetical vignettes, revealed that the perception of shared condemnation was positively correlated with moral outrage and moral disgust, and negatively with empathy for violators. Study 2 examined the causal relationship between perceived shared condemnation and moral emotions by experimentally manipulating the perception of shared condemnation. In particular, participants in Study 2 were exposed to information indicating that the majority of previous participants either had condemned or had not condemned particular moral violations. In support of the causal hypothesis, the experimentally increased perception of shared condemnation amplified moral outrage. To confirm the external validity of this finding, we conducted an autobiographical recall study (Study 3), whereby respondents reported a recent incident where they had witnessed someone’s immoral behaviour. Again, perceived shared condemnation was positively correlated with moral outrage and moral disgust.

## Results

### Study 1

We prepared 30 hypothetical norm violation scenarios (see Table S1), two of which were adapted from a previous study^45^. The 30 scenarios, divided into two sets of 15 scenarios, varied in terms of outcome severity and moral domain (e.g., loyalty to one’s in-group, purity). None of the 30 scenarios involved readily identifiable victims, as it is known that empathic concern for victims causes vicarious anger, which is conceptually distinct from moral outrage^46^. Examples of the scenarios include *Person A downloaded a large amount of music and movies for free from an online file sharing site*; *Person A attended a wedding ceremony wearing everyday clothes even though he/she knew that it was inappropriate*; and *Person A, who is an entrepreneur, moved his/her cooperate bank account to a foreign bank for the purpose of avoiding taxation*. A total of 237 Japanese undergraduate students were exposed to one of two sets of 15 scenarios, and rated their emotional reactions (moral outrage, moral disgust, empathy for the depicted violator), perceived shared condemnation (*What proportion of Japanese citizens do you think would condemn Person A?*), and willingness to inflict two types of punishment. Two types of punitive intent were measured using hypothetical vignettes: The first vignette (henceforth referred to as ‘wallet’) was as follows: *You happen to witness Person A drop his/her wallet. He/she has not yet gone far away. Would you tell him/her about the wallet?* Not telling Person A about the wallet was interpreted as an informal form of punishment. The second vignette (henceforth referred to as ‘fine’) was as follows: *You are entitled to impose a fine on Person A. Would you impose a fine on Person A?*

**Figure 1:**
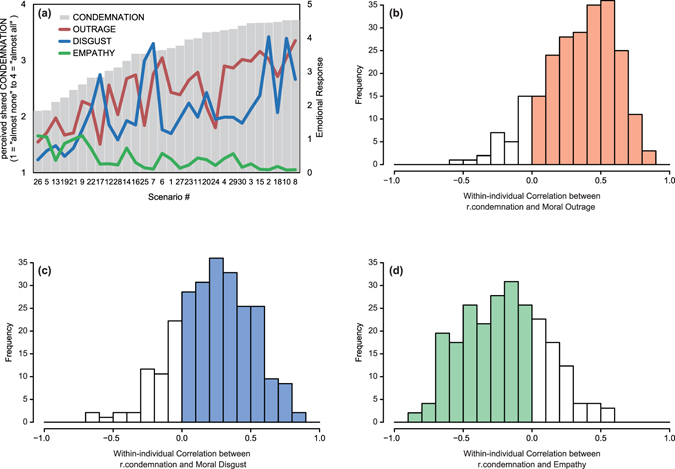
Relationship between Perceived Shared Condemnation and Moral Emotions in Study 1. (**a**) The scenarios were ordered by the level of CONDEMNATION_*k*_ (across-participants average of perceived shared condemnation for each violation scenario). The CONDEMNATION_*k*_ scores are shown by grey bars. The red line shows OUTRAGE_*k*_. The blue line shows DISGUST_*k*_. The green line shows EMPATHY_*k*_. (**b**) The distribution of the within-individual correlation between r.condemnation_*jk*_ (=*j*-th participants perceived shared condemnation of the *k*-th violation – CONDEMNATION_*k*_) and moral outrage. The red bars indicate positive *r*s between r.condemnation_*jk*_ and moral outrage for most participants. (**c**) The distribution of the within-individual correlation between r.condemnation_*jk*_ and moral disgust. (**d**) The distribution of the within-individual correlation between r.condemnation_*jk*_ and empathy.

For each scenario, the reported moral emotions and perceived shared condemnation were aggregated across participants. These aggregated variables are henceforth denoted as OUTRAGE_*k*_, DISGUST_*k*_, EMPATHY_*k*_, and CONDEMNATION_*k*_ (the subscript *k* corresponds to the scenario, and thus ranges from 1 to 30). For Study 1, the uppercased variables designate scenario-level variables. In Fig. 1a, the 30 scenarios are ordered by level of average condemnation: from less consensually condemned (left) to more condemned violations (right). Each grey bar in Fig. 1a indicates the level of CONDEMNATION_*k*_, and the red, blue, and green lines indicate the levels of OUTRAGE_*k*_, DISGUST_*k*_, and EMPATHY_*k*_, respectively. As can be seen in Fig. 1a, CONDEMNATION was significantly correlated with OUTRAGE, DISGUST, and reduced EMPATHY: *r*_28_ = 0.78, 0.58, and −0.74, respectively (all *P*s < 0.001). These significant correlations indicate that more consensually condemned norm violations, on average, induced greater moral outrage and disgust, and diminished empathy for the violator.

More central to our interest, we tested whether perceived shared condemnation would predict moral emotions in each participant even after controlling for the effect of the consensual component (i.e. CONDEMNATION_*k*_). The hierarchical linear model approach^47^ was employed to test this effect because 15 condemnation and moral emotion scores were nested within each participant (see SI Study 1 Method and Results for the full descriptions of the models and Tables S2, S3, and S4 for the results). The results indicated that even after controlling for the scenario effect (CONDEMNATION_*k*_) and other potentially confounding variables (e.g., sex), condemnation significantly predicted moral emotions. In the main text, however, we opted to use the following more intuitive presentations. To remove the effect of CONDEMNATION_*k*_, we subtracted CONDEMNATION_*k*_ from each participant’s perceived shared condemnation score (i.e. condemnation_*jk*_, which is *j*-th participant’s perceived shared condemnation associated with scenario *k*). This remainder score is referred to as r.condemnation_*jk*_. Positive (negative) values of r.condemnation_*jk*_ indicate that the focal participant, as compared to the other participants in this study, overestimated (underestimated) the proportion of Japanese citizens (i.e., reference group) who would condemn a specific norm violation. As 15 r.condemnation_*jk*_ and moral emotion scores were nested within each participant, we computed the correlation coefficient between 15 r.condemnation_*jk*_ and 15 moral emotion scores for each individual (i.e., for each level of *j*). Figs. 1b, c and d show the distributions of these within-individual correlations for moral outrage, moral disgust, and empathy. The within-individual correlations between r.condemnation_*jk*_ and moral outrage/disgust were mostly positive (see coloured bars in Figs. 1b and c) and the within-individual correlations between r.condemnation_*jk*_ and empathy were mostly negative (see coloured bars in Fig. 1d). Therefore, it can be said that each individual’s perceived shared condemnation predicted the intensity of moral emotions above and beyond the actually shared component (CONDEMNATION_*k*_).

We then examined which of the three moral emotions would predict two types of punitive behaviours (i.e. ‘wallet’ and ‘fine’). A series of hierarchical linear models was employed to test the relationship between each participant’s 15 punitive intention scores and moral emotion scores (moral outrage, moral disgust, and reduced empathy). All three moral emotions significantly predicted unwillingness to tell the violator about the dropped wallet (Table S6), whereas only moral outrage significantly predicted willingness to impose a fine (Table S5).

### Study 2

Confirming the significant correlation between perceived shared condemnation and moral emotions, we proceeded to test causality. Participants (102 undergraduate students) were presented six scenarios that were associated with either medium (around 2.5 on a scale ranging from 0 to 5) or low (around 1.0) levels of moral outrage in Study 1. Each scenario was accompanied by either information indicating that the vast majority of previous participants in a similar study had considered the depicted violation ‘extremely bad’ (high shared condemnation condition), information indicating that the majority of previous participants had considered the violation ‘not terribly bad’ (low shared condemnation condition), or no information about the previous participants’ opinions (see Fig. S1 for the information given to participants). Each scenario was followed by the same measures used in Study 1 (i.e., emotional reactions, perceived shared condemnation, and punitive intentions). The experimenter explicitly told participants that the information was presented just for their reference, and they should not conform to the previous participants’ opinions.

**Figure 2:**
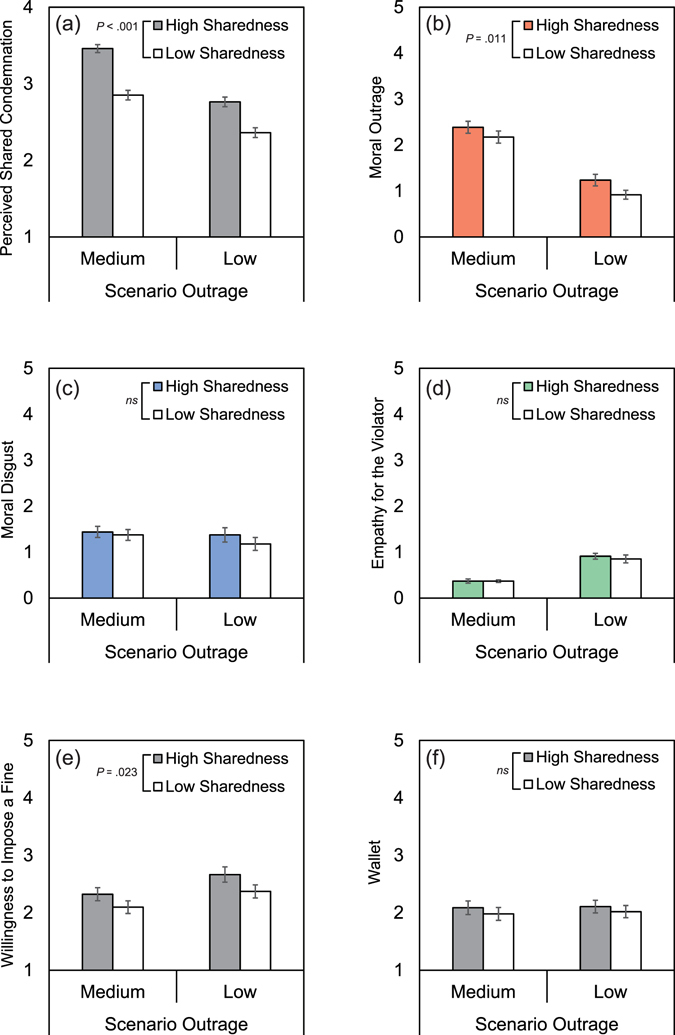
Mean Dependent Variables in Study 2 as a Function of Scenario Outrage (Medium vs. Low) and Information about Others’ Condemnation (High vs. Low). The dependent variables were (**a**) perceived shared condemnation (i.e., manipulation check item in Study 2), (**b**) moral outrage, (**c**) moral disgust, (**d**) empathy, (**e**) willingness to impose a fine, and (**f**) unwillingness to tell about the dropped wallet.

The perceived shared condemnation measure served as the manipulation check in Study 2. As shown in Fig. 2a, the scenario outrage level (medium vs. low) had a significant effect: Participants expected greater sharedness of condemnation for the medium outrage scenarios than for the low outrage scenario (*F*_1, 101_ = 102.48, *P* < 0.001, ${{{\rm{\eta }}}_{{\rm{G}}}}^{2}$ = 0.24). More importantly, participants also expected greater sharedness of condemnation in the high shared condemnation condition than in the low shared condemnation condition (*F*_1, 101_ = 65.25, *P* < 0.001, ${{{\rm{\eta }}}_{{\rm{G}}}}^{2}$ = 0.18). Therefore, the manipulation was successful.

We then tested whether the high shared condemnation information amplified the moral emotions by a 2 (information: high vs. low shared condemnation) × 2 (scenario outrage: medium vs. low) analysis of variance (ANOVA). As shown in Fig. 2b, the main effects of information (*F*_1, 101_ = 6.79, *P* = 0.011, ${{{\rm{\eta }}}_{{\rm{G}}}}^{2}$ = 0.024) and scenario outrage (*F*_1, 101_ = 136.24, *P* < 0.001, ${{{\rm{\eta }}}_{{\rm{G}}}}^{2}$ = 0.332) were significant for moral outrage (Table S7). However, the effect of information on moral disgust and empathy was not significant (Fig. 2c and d, see also Table S7). For practical reasons, we only controlled for the level of moral outrage in choosing the six scenarios used in Study 2. This might have diluted the effect of information on the other two emotions. In addition, the two punitive intention scores (‘wallet’ and ‘fine’) were submitted to comparable ANOVAs. As shown in Fig. 2e, the shared condemnation information significantly increased willingness to impose a fine on the violator (*F*_1, 101_ = 5.29, *P* = 0.023, ${{{\rm{\eta }}}_{{\rm{G}}}}^{2}$ = 0.021), but the effect of information was not significant for the wallet version of punishment (Fig. 2f, and see Table S8).

### Study 3

Studies 1 and 2 employed hypothetical scenarios, and revealed that the intensity of moral emotions was modulated by perceived shared condemnation. To confirm the external validity, an online survey (Study 3) was conducted. In Study 3, 687 Japanese citizens reported their real-life experiences of witnessing moral violations. To examine third-party reactions to norm violations, respondents were explicitly told to report a violation in which they themselves had not been directly involved.

The online survey comprised five sections in addition to a screening section: (i) description of the violation in an open-ended format (characteristics of the reported violations are summarized in Table S9); (ii) victim(s)—the presence/absence of a victim/victims, (if a victim was present) relationship with the victim, and emotional responses to the victim; (iii) norm violator(s)—violator type (individual or group), relationship with the violator, and emotional reactions to the violator; (iv) perceived shared condemnation and indirect damage to respondents themselves; and (v) intervention and motivations underlying the intervention (for details of the survey items, see SI Study 3 Method). Demographic information (e.g., sex, age) was collected in the screening section.

**Figure 3:**
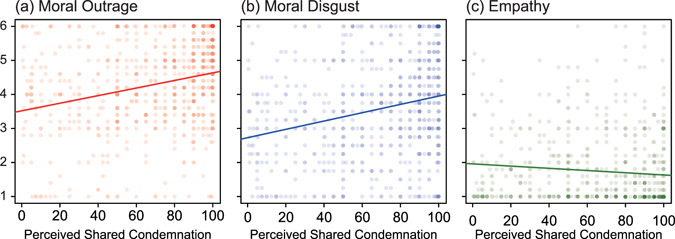
Scatter Plots Showing the Relationship between Perceived Shared Condemnation and Three Moral Emotions in Study 3. (**a**) Moral outrage, (**b**) moral disgust, and (**c**) empathy.

In Study 3, respondents estimated what proportions of Japanese citizens and their friends would condemn the violation they had witnessed. The two estimates (one for the Japanese citizens and one for their friends) were highly correlated with each other (*r* = 0.72), and thus aggregated as the single perceived shared condemnation score. As shown in Figs. 3a to c (see also Table S10), perceived shared condemnation was positively correlated with moral outrage (*r*_685_ = 0.27, *P* < 0.001) and moral disgust (*r*_685_ = 0.25, *P* < 0.001), and negatively with empathy for the violator (*r*_685_ = −0.09, *P* = 0.014). The correlations with moral outrage, moral disgust, and empathy remained significant after controlling for respondents’ sex and age, the presence of victims (either individual or collective), and indirect damage to self in a series of multiple regression analyses: βs = 0.23, 0.22, and −0.10 for moral outrage (*P* < 0.001), moral disgust (*P* < 0.001), and empathy (*P* = 0.010), respectively. A similar pattern emerged when we focused on violations that involved an individual victim (Table S12) and on those that did not involve any victim (Table S13)—perceived shared condemnation consistently predicted moral outrage and moral disgust.

We then examined whether moral emotions would predict respondents’ intervention behaviours. We conducted two separate logistic regression analyses with intervention as a dichotomous dependent variable, and moral emotions, respondents’ sex and age, the presence of victims (either individual or collective), and indirect damage to self as the predictor variables. We did not enter moral outrage and moral disgust in a single model because they were extremely highly correlated (*r* = 0.71, see also Table S10) and caused the multicollinearity problem. The results showed that moral outrage and moral disgust significantly increased the probability of intervention (odds ratios = 1.35 and 1.26 for moral outrage and moral disgust, respectively). Empathy for the violator significantly decreased the probability of intervention (odds ratio was approximately 0.70 in the two models; see Table S14 for more details).

Close scrutiny of the reported interventions revealed that many interventions (e.g., calling the police) did not qualify as costly third-party punishment. Respondents who had intervened in the violation rated the importance of three plausible motivations of their intervention on a five-point scale (1 = ‘not at all’ to 5 = ‘very much’): motivations to punish the violator, compensate the victim, and restore fairness/justice. A 2 (sex) × 3 (motivation) ANOVA involving the latter factor as repeated measures yielded a significant main effect of motivation (*F*_2, 248_ = 7.61, *P* < 0.001, η_p_^2^ = 0.058). Motivation to restore fairness/justice (3.77 ± 1.15) was significantly more important than motivations to punish the violator (3.32 ± 1.41; *t*_248_ = 3.07, *P* = 0.002) and compensate the victim (3.24 ± 1.46; *t*_248_ = 3.62, *P* < 0.001). Therefore, we should admit that not all interventions were driven by punitive motivation. Nevertheless, it is worth reporting that moral outrage and moral disgust were significantly correlated with punitive motivation (*r*_124_ = 0.29, *P* = 0.001 for moral outrage, and *r*_124_ = 0.29, *P* = 0.001 for moral disgust; see Table S15). Empathy was negatively correlated with the punitive motivation, but did not reach the significance level (*r*_124_ = −0.14, *P* = 0.131). Compensatory motivation was not significantly correlated with any of the three moral emotions, and fairness/justice motivation was significantly correlated only with moral outrage (*r*_124_ = 0.20, *P* = 0.022). The stronger association between moral emotions and punitive motivation seems to suggest that moral emotions drive people to engage in punitive intervention.

## Discussion

The three studies revealed that perceived shared condemnation modulated moral emotions. When participants expected that others would condemn a particular norm violation, this enhanced moral outrage and moral disgust, while it decreased their empathy for the violator. Study 1 examined this association using 30 hypothetical scenarios. First, at the aggregated level, as shown in Fig. 1a, perceived shared condemnation (CONDEMNATION_*k*_) was positively correlated with moral outrage and moral disgust (OUTRAGE_*k*_ and DISGUST_*k*_), and negatively correlated with empathy (EMPATHY_*k*_). Moreover, the correlations between perceived shared condemnation and moral emotions were observed above and beyond the aggregated level—each participant’s unique perception of shared condemnation (r.condemnation_*jk*_) was correlated positively with moral outrage and moral disgust (Figs. 1b and c), and negatively with empathy for the violator (Fig. 1d). In Study 2, the perception of shared condemnation was experimentally manipulated. Increased belief in shared condemnation amplified moral outrage. By examining respondents’ real-life experiences of witnessing a moral violation, Study 3 was aimed at confirming the external validity of the finding. The results demonstrated that people were more outraged at violations that they had expected many others would condemn. Moreover, these three studies consistently showed that moral emotions promoted some form of punishment (e.g., imposing a hypothetical fine) or intervention (e.g., calling the police).

This research used the perception of whether others condemn a norm violation, as opposed to being simply angry at it, as an independent variable. Notice that the act of condemning someone (i.e., a behaviour) is more conspicuous and unambiguous than the act of feeling an emotion. Such conspicuousness is important because extant models of coordinated punishment (or conditional punishment) assume that punishers can somehow be aware of the frequency of other punishers around them^24, 25^. One possible process is that conspicuous condemnation leads to the coordination of punishment in the following manner: There are a sufficient number of community members who openly condemn a particular norm violation at the outset. Observing these initial condemnations, other community members may become increasingly angrier and more willing to partake in the punishment. In contrast, if there is not a sufficiently large group of initial condemnations, the condemners may fail to recruit fellow punishers and eventually abandon the infliction of a punishment. This process can result in coordinated punishment. Notice that this process may also facilitate the evolution of punishment by allowing punishers to inflict punishment only when they can reduce norm violators’ payoffs to a value relatively lower than their own^48^.

One might pose a question concerning whether the observed level of correlation would be sufficient for coordinating punishment. We admit that the reported correlations at the individual level were modest at best. However, it is noteworthy that the reported data were collected in Japan, a modernized country where many traditional norms have lost their importance. It is expected that in relatively closed communities or small-scale societies, norms are more widely shared and exert stronger influences on community members’ behaviours than in Japan^22, 23^. Socially well-shared norms may also serve as a coordination device^27, 28^ and bolster the correlation between perceived shared condemnation and moral emotions. Moreover, in small-scale societies, gossiping might accentuate an initial small correlation via mutual confirmation of shared condemnation and make it more effective for coordinating punishment^23, 49^. It is interesting to systematically observe how the dynamic processes triggered by perception of shared condemnation (e.g., mutual confirmation via gossiping, modulation of moral emotions) leads to coordinated punishment in relatively closed communities and small-scale societies.

One limitation of this research was that none of the three studies involved behavioural measures of punishment^50^. As a partial remedy, in Study 3, we asked respondents whether they had somehow intervened in the violation. However, such real-world behaviours are constrained by various factors and do not necessarily reflect actors’ motivations. Behavioural measures must be included in future studies. The third-party punishment game is an obvious option. In addition, other subtle measures of punitive motivations have recently been proposed^51^. By combining these research methods, future studies should examine whether the perception of shared condemnation and resultant shared moral emotions in fact facilitate coordinated punishment. Recall that coordinated punishment was proposed as an alternative model to uncoordinated (individually inflicted) punishment^24, 25^. However, there are other evolutionarily viable ways to inflict punishment, such as institutionalising (or centralising) punishment^52^, punishing all members of a group in which at least one member has cheated^53^, and probabilistically inflicting punishments^54, 55^. In addition to punishing norm violators, it is also possible for cooperative members to exclude norm violators from their groups so that they cannot free-ride on others’ contributions^56^. Inclinations towards each of these strategies—as they related to moral emotions—need to be empirically examined. For example, moral disgust may be more strongly associated with ‘exclusion’ or withdrawal from interactions with norm violators (e.g., not telling about the dropped wallet) than with active forms of punishment (e.g., imposing a fine). Behavioural experiments are suitable to test the correspondence between strategies implicated in theoretical models and people’s actual punitive behaviour.

In future behavioural experiments, it may also be important to take into account the frequency and severity of punishments so that researchers can evaluate whether shared moral emotions actually lead to optimal levels of punishment^54, 55, 57^. This may be of practical importance. In the age of the Internet, it has become much easier to be exposed to the opinions of various people, including small fractions of people who strongly condemn someone’s perceived misbehaviour. An initially small group of condemners might be joined by those who happened to be exposed to the condemnation, and the group may increase in visibility and influence. Such a process could escalate shared condemnation (or computer-mediated punishment) beyond the optimal level, which may help explain recent social phenomena such as social media witch hunts and online shaming. Behavioural experiments informed by evolutionary models may reveal under what conditions such escalation is likely/unlikely to occur.

## Methods

### Study 1

Participants were 237 undergraduates at two Japanese universities (104 males, 132 females, and one unreported; mean age ± s.d. = 19.71 ± 1.45 years). Participants were given a questionnaire packet including 15 norm violation scenarios (30 scenarios randomly split into two sets) and the Moral Foundations Questionnaire (MFQ)^58^. Each of the 15 norm violation scenarios was accompanied by the measures of emotional reactions, perceived shared condemnation, and willingness to punish the violator (for details, see SI Study 1 Method). Moral outage (e.g., angry, indignant) and empathy (e.g., warm, compassionate) were each measured by five items adapted from Batson and colleagues’ experiment^46^. For moral disgust, four items (e.g., disgusting, repulsive) were written by the authors. Emotional reactions were measured on a 6-point scale (0 to 5); perceived shared condemnation was measured on a 4-point scale; and punitive intention was measured on a 5-point scale.

### Study 2

Participants were 102 undergraduate students at a Japanese university (40 males and 62 females; mean age ± s.d. = 18.96 ± 0.70 years old). Participants were presented six norm violation scenarios (Scenarios 1, 12, 17, 19, 21, and 23 in Table S1), each of which was accompanied by the same measures used in Study 1. Three of the six scenarios were associated with medium levels of moral outrage in Study 1 (mean ± s.d. = 2.34 ± 1.43, 2.60 ± 1.50, and 2.75 ± 1.53 for Scenarios 1, 12, and 23, respectively), and the other three were associated with low levels of moral outrage (mean ± s.d. = 0.85 ± 1.00, 1.12 ± 1.16, and 1.18 ± 1.11 for Scenarios 17, 19, and 21, respectively). We excluded scenarios that had elicited intense moral outrage in Study 1 to avoid the ceiling effect. The scenarios were ordered alternating the medium- and low-level outrage scenarios. Of the six scenarios, two (one medium outrage scenario and one low outrage scenario) were accompanied by information indicating high levels of shared condemnation. Another two scenarios (one medium outrage scenario and one low outrage scenario) were accompanied by information indicating low levels of shared condemnation (see Fig. S1 for the information presented to participants). The remaining two scenarios were not accompanied by any information. The order of the information was either high-high-no-no-low-low or low-low-no-no-high-high, and the order of the scenarios was counterbalanced by the Latin square method. The middle two scenarios, which were not accompanied by any information, served to mitigate possible carryover effects.

### Study 3

Respondents were recruited via an online survey service provided by a Japanese online research company, Cross Marketing Inc. A total of 834 respondents (421 males and 413 females; mean age ± s.d. = 44.17 ± 14.51 years old) completed the survey. However, 147 respondents did not follow the instructions (e.g., reporting that they could not remember any such incident; reporting an incident in which they themselves were directly harmed). Accordingly, responses from 687 respondents were retained in the subsequent data analyses (see SI Study 3 Method for more details of the items in the survey).

### Data Availability

The data sets from the three studies are uploaded as supplementary files of this article.

### Ethics Statements

The present research was approved by the Human Research Ethics Committee of the Graduate School of Humanities, Kobe University. We conducted this research in compliance with the principles of the Declaration of Helsinki. All participants in Study 1 expressed their consent to the participation by completing the questionnaire. All participants in Study 2 provided a written informed consent. Respondents in Study 3 electronically consented to the participation and voluntarily completed the survey.

## Electronic supplementary material

Supplementary Dataset

Supplementary Information
