# Supplementary material for: Perceived Shared Condemnation Intensifies Punitive Moral Emotions
Source: Sci Rep. 2017 Aug 4;7:7289. doi: 10.1038/s41598-017-07916-z (PMC5544740; doi:10.1038/s41598-017-07916-z)
Supplement: Supplementary file 2 — Supplementary Information [file 41598_2017_7916_MOESM2_ESM.pdf]

Supplementary Information (Method, Materials, and Additional Analyses)

Perceived Shared Condemnation Intensifies Punitive Moral Emotions

Study 1

Method

Participants. Participants were 237 undergraduates at two Japanese universities (104 males, 132 females, and one unreported; mean age ± s.d. = 19.71 ± 1.45 years).

Materials. Participants were given a questionnaire packet including 15 norm violation scenarios and the Moral Foundations Questionnaire (MFQ)[58] developed by Graham et al. Participants completed the questionnaire at their own pace.

Scenarios. We prepared 30 norm violation scenarios, none of which included a readily identifiable victim (see Table S1 for a list of the 30 scenarios). We decided not to include readily identifiable victims in the scenarios because the presence of victims might cause empathic anger, which is elicited by harm against a cared-for other[44], but not anger at the norm violation per se. As a result, most of the 30 scenarios mapped onto four of the five fundamental domains of morality[49]: fairness, loyalty, authority, and purity. Owing to the time constraint, we divided the 30 scenarios randomly into two sets (Sets A and B). Set A comprised Scenarios 1 – 15 in Table S1, and Set B comprised Scenarios 16 – 30 in Table S1. In addition, to reduce possible order effects, we created two versions of each set by randomly ordering 15 scenarios. Participants received a questionnaire containing either Set A1, A2, B1 or B2. They were presented 15 norm violation scenarios and asked to rate their emotional reactions, perceived illegality, perceived shared condemnation, impressions of the action/actor, and punitive attitudes.

Emotional Reactions. Three emotional reactions—moral outrage, moral disgust, and reduced empathy—were measured by 16 items: five items for moral outrage (i.e., angry, indignant, mad, outraged, perturbed), four items for moral disgust (i.e., disgusting, repulsive, impure, makes my skin crawl), five items for empathy (i.e., sympathetic, compassionate, warm, tender, softhearted), and two filler items (i.e., surprise, strange). The moral outrage and empathy items were adapted from Batson et al.'s study[46]. To accommodate the items in Japanese, we omitted four outrage items and one empathy item from Batson et al.'s original study. We wrote the disgust items by ourselves. These emotional reaction items were rated on a 6-point scale (0 = 'do not feel at all' to 5 = 'feel it very much'). We computed Cronbach's α coefficients for the 30 scenarios separately. The mean (± s.d.) Cronbach's α coefficients were .93 ± .017, .85 ± .038, and .76 ± .100 for moral outrage, moral disgust, and empathy, respectively. For each participant, we computed 15 outrage, 15 disgust, and 15 empathy scores.

Perceived Shared Condemnation. Perceived shared condemnation was measured by a single item: *What proportion of Japanese citizens do you think would condemn Person A?* (1 = 'almost none' to 4 = 'almost all'). This shared condemnation rating was likely influenced by participants' belief in the illegality of the target violation. Therefore, we also measured perceived illegality: *Do you think that Person A's behaviour violated Japanese law?* In addition, we measured perceived wrongness of the violations, and the impression of Person A: *How bad do you think Person A's behaviour is?; Do you think that Person A is a likeable person?* These items were rated on a 4-point scale (1 = 'completely disagree' and 4 = 'strongly agree').

Table S1 | List of Scenarios Used in Study 1 and Correlation Coefficients between Participants' Badness Judgment of Each Scenario and the Five Moral Concerns Measured by the Moral Foundations Questionnaire.

	Moral Foundations (Cronbach's α Coefficient)	C (.66)	F (.59)	L (.55)	A (.56)	P (.51)
1	Person A attended a wedding ceremony wearing everyday clothes even though he/she knew that it was inappropriate.	.17	.05	.23*	.16	.20*
2	Person A accidently ran over his/her dog, thereby killing it. After that he/she cooked and ate the dog. †	.30**	.08	.27**	.03	.19*
3	Federal employee A divulged classified information, which he/she had accidentally uncovered, to another country.	.15	.10	.13	.06	.22*
4	Person A, who is an entrepreneur, moved his/her cooperate bank account to a foreign bank for the purpose of avoiding taxation. So he/she did not pay corporate tax.	.16	.08	.26**	.10	.25**
5	Person A, who is a local official, is repeatedly treated to dinner by members of a firm that he/she is responsible for overseeing.	−.13	−.06	−.10	−.12	−.23*
6	Person A, who was riding on a crowded train, nonetheless saved a seat for his/her friend who wouldn't board the train until a few stops later.	.13	.08	.06	.05	.15
7	Person A scraped the shell off a living insect, and uploaded a video of this to YouTube.	.31**	.23*	.12	.06	.19*
8	Person A entered an empty Buddhist temple in the middle of the night, knocked down six Buddhist statues, and covered them in spray-paint.	.08	.19*	−.02	.16	.08
9	Person A got pregnant with someone from work, but she got an abortion because both she and the co-worker were facing financial struggles.	.18	.16	.18*	.06	.25**
10	Person A, who is a necrophiliac, works as an undertaker. Prior to cremation, he enjoys secretly photographing corpses.	.30**	.09	.22*	.16	.21*
11	Person A drove to work by car while still intoxicated from last night's drinking. Fortunately, he/she did not cause an accident.	.28**	.11	.17	.11	.05

12	Person A purposely sung the national anthem in a loud and annoying voice at his/her college entrance ceremony.	.17	.08	.24*	.23*	.22*
13	Person A consistently lies about the date of his/her own birthday whenever he/she is asked.	.29**	.21*	.21*	.07	.27**
14	Person A overslept and missed an exam. He/she then lied about the reason for having missed the exam, namely that his/her grandma died. He/she later took and passed the exam.	.19*	.13	.22*	.08	.17
15	Person A, as a sort of practical joke, threatened to blow up an elementary school in an online message. The school was subsequently evacuated for a number of days.	.25**	.23*	.04	.05	.15
16	Person A, in order to reserve a spot at a public fireworks display, spray-painted a coloured line in the grass.	−.00	.08	.03	.03	.07
17	Person A ate a chicken carcass he had recently used for masturbation.†	.28**	.22*	.27**	.31**	.30**
18	Person A, a computer engineer, accessed the website of the Japanese Ministry of Defence, and altered the content in order to discredit Japan.	.17	.12	.19*	.16	.02
19	Person A, an entrepreneur, moved his/her head office to a foreign country for the purpose of avoiding local taxation. So he/she did not pay taxes in Japan.	.21*	.18	.13	.17	.23*
20	Person A, a university student, wanted to make this a summer to remember, and ran naked through the streets at midnight, while his/her friend filmed the commotion.	.04	.02	.20*	.23*	.13
21	Person A downloaded a large amount of music and movies for free from an online file sharing site.	.18	.11	.17	.34**	.30**
22	Person A was swimming in the ocean at a public beach. He/she felt the need to pee, and so he/she did.	−.07	.06	.25**	.03	.14
23	Person A thought his/her family’s Buddhist altar and religious relics, each of which symbolizes his/her deceased ancestors, were a hindrance, so he/she threw them in the trash.	.13	.10	.13	.15	.02
24	Person A grows marijuana in his/her garden for personal use. He/she has never given nor sold this marijuana, and has no intention to do so in the future.	.14	.08	.21*	.41**	.36**

25	Person A, who is a necrophiliac, uses a picture of a dead body as his/her desktop wallpaper.	.34**	.23*	.23*	.10	.27**
26	Person A always jaywalks when there are no cars around.	−.00	.07	.15	.09	.09
27	Person A took a movie of him/herself burning the national flag of Japan, and uploaded this video to YouTube.	.10	.08	.39**	.21*	.24**
28	Person A frequently wears a homemade police uniform and walks the streets pretending to be a genuine police officer.	.10	.15	.02	.18*	.04
29	Person A decided it would be too difficult to pass an exam on his/her own, so he/she used a cheat sheet in order to pass.	.13	.23*	.05	.12	.02
30	Person A, as a sort of practical joke, created a computer virus that displays useless symbols on a computer's screen. Many computers were infected due to the virus, and many people had to waste their time removing the virus.	.20*	.26**	.27**	.29**	.18*

Notes. In the Moral Foundations rows, 'C', 'F', 'L', 'A', and 'P' represents 'care', 'fairness', 'loyalty', 'authority', and 'purity', respectively.

† These scenarios were adapted from Haidt, Koller, and Dais's paper[45].

Willingness to Inflict Punishment. In addition, participants answered about their willingness to informally or formally punish Person A. Two items were as follows: (i) *You happen to witness Person A drop his/her wallet. He/she has not yet gone far away. Would you tell him/her about the wallet?* (ii) *You are entitled to impose a fine on Person A. Would you impose a fine on Person A?* These two items were rated on a 5-point scale (1 = 'never do so' to 5 = 'absolutely do so'). The responses to the wallet item were reverse-coded.

Moral Foundations Questionnaire. These scenarios were followed by the MFQ. The MFQ comprises 30 items to measure participants' valuations of five domains of morality (i.e., care, fairness, loyalty, authority, purity). Six items are associated with each of the five domains. The MFQ was included in this study for exploratory purposes.

Results

We tested whether perceived shared condemnation would predict moral emotions after controlling for the actual shared component of condemnation (CODEMNATION). As explained in the main text, for each scenario, perceived condemnation scores were aggregated across participants. This aggregated variable, CONDEMNATION_k, approximates the actual shared level of condemnation directed at the *k*-th violation. The three figures in the main text imply that each individual's unique perception of shared condemnation (i.e., perceived shared condemnation after controlling for aggregated CONDEMNATION_k) was positively associated with moral outrage and moral disgust (Figs. 1b and 1c, respectively), and negatively correlated with empathy (Fig. 1d).

The above patterns were formally tested using the hierarchical linear modelling approach because 15 scores were nested within individuals. We tested the effects of perceived shared condemnation on moral outrage, disgust, and empathy controlling for CONDEMNATION_k. In

addition, we included the within-participant mean of perceived shared condemnation, which corresponds to the *j*-th participant's bias in estimating others' level of condemnation. We refer to this within-participant mean as 'biased perception$_j$'. Participants with high biased perception scores tended to think that others would strongly condemn various types of norm violations, whereas participants with low biased perception scores tended not to think that others would strongly condemn norm violations. Each individual had 15 perceived shared condemnation scores (i.e. the perceived shared condemnation scores were nested within each individual). At the same time, each score was associated with a particular scenario (i.e. the perceived shared condemnation scores were also nested within each scenario). Therefore, we used the cross-classified random effect model (HCM2) in HML7 (Scientific Software International)[47]. We tested two types of models. The first model (Model 1) included only perceived shared condemnation-related variables (i.e., perceived shared condemnation$_{ijk}$, CONDEMNATION$_k$, biased perception$_j$). We also tested models including participants' sex and university (data were collected at two universities). The specific models that we tested are as follows (we follow the notations used by HLM, and use Greek letters although we did not standardize the coefficients):

Model 1

Level 1

$$Y_{ijk} = \pi_{0jk} + \pi_{1jk}(\text{perceived shared condemnation}_{ijk}) + e_{ijk}$$

where Y_{ijk} = individual *j*'s moral emotions (moral outrage, moral disgust, or empathy) for scenario *k*.

Level 2

$$\pi_{0jk} = \theta_0 + b_{00j} + c_{00k} + \gamma_{01}(\text{biased perception}_j) + \beta_{02}(\text{CONDEMNATION}_k)$$

$$\pi_{1jk} = \theta_1$$

Model 2

Level 1

$$Y_{ijk} = \pi_{0jk} + \pi_{1jk}(\text{perceived shared condemnation}_{ijk}) + e_{ijk}$$

Level 2

$$\pi_{0jk} = \theta_0 + b_{00j} + c_{00k} + \gamma_{01}(\text{sex}_j) + \gamma_{02}(\text{university}_j)$$
$$+\ \gamma_{01}(\text{biased perception}_j) + \beta_{04}(\text{CONDEMNATION}_k)$$

$$\pi_{1jk} = \theta_1 + \gamma_{11}(\text{sex}_j) + \gamma_{12}(\text{university}_j)$$

where sex was coded as 0 = female and 1 = male.

The results of the series of hierarchical linear models are summarized in Tables S2, S3, and S4 for moral outrage, moral disgust, and reduced empathy, respectively. Confirming the trends in Fig. 1a, CONDEMNATION predicted moral outrage and diminished empathy for the violator (see the rows of β_{02} and β_{04} in Models 1 and 2, respectively). The effect of CONDEMNATION on moral disgust was marginally significant. More importantly, corroborating the patterns depicted in Figs. 1b to 1d, even after controlling for the effect of CONDEMNATION, perceived shared condemnation was significantly positively associated with moral outrage and moral disgust, and negatively with empathy for the perpetrator (see the rows of θ_1 in Models 1 and 2).

Table S2 | Summary of Hierarchical Linear Model Estimations: Tests of the Effect of Perceived Shared Condemnation, Within-Scenario Average Condemnation ($CONDEMNATION_k$), Within-Individual Average Condemnation ($Perception Bias_j$), and Other Control Variables on Moral Outrage

Moral Outrage	Coefficients		Standard Error	$\|t\|$	*df*	*p*-value
Model 1						
π_0						
Intercept θ_0	−3.84	***	0.807	4.76	3271	< .001
Perception Bias γ_{01}	0.66	***	0.183	3.60	234	< .001
CONDEMNATION β_{02}	0.56	**	0.184	3.06	28	.005
Perceived Shared Condemnation π_1						
Intercept θ_1	0.79	***	0.029	27.29	3271	< .001
Model 2						
π_0						
Intercept θ_0	−3.74	***	0.813	4.60	3269	< .001
Sex γ_{01}	−0.06		0.167	0.34	234	.735
University γ_{02}	−0.25		0.171	1.43	234	.153
Perception Bias γ_{03}	0.66	***	0.185	3.58	234	< .001
CONDEMNATION β_{04}	0.56	**	0.184	3.06	28	.005
Perceived Shared Condemnation π_1						
Intercept θ_1	0.75	***	0.038	19.48	3269	< .001
Sex γ_{11}	0.02		0.044	0.47	3269	.639
University γ_{12}	0.08	+	0.045	1.69	3269	.091

Table S3 | Summary of Hierarchical Linear Model Estimations: Tests of the Effect of Perceived Shared Condemnation, Within-Scenario Average Condemnation (CONDEMNATION$_k$), Within-Individual Average Condemnation (Perception Bias$_j$), and Other Control Variables on Moral Disgust

Moral Disgust	Coefficients		Standard Error	|*t*|	*df*	*p*-value
Model 1						
π_0						
Intercept θ_0	−3.04	**	1.106	2.75	3271	.006
Perception Bias γ_{01}	0.45	*	0.206	2.19	234	.029
CONDEMNATION β_{02}	0.56	$^+$	0.287	1.95	28	.061
Perceived Shared Condemnation π_1						
Intercept θ_1	0.55	***	0.027	20.49	3271	< .001
Model 2						
π_0						
Intercept θ_0	−2.82	*	1.104	2.56	3269	.011
Sex γ_{01}	−0.17		0.167	1.03	234	.303
University γ_{02}	−0.13		0.171	0.77	234	.442
Perception Bias γ_{03}	0.42	*	0.206	2.04	234	.043
CONDEMNATION β_{04}	0.56	$^+$	0.286	1.96	28	.061
Perceived Shared Condemnation π_1						
Intercept θ_1	.53	***	.036	14.65	3269	< .001
Sex γ_{11}	.08	*	.041	2.06	3269	.040
University γ_{12}	−0.03		.042	0.74	3269	.461

Table S4 | Summary of Hierarchical Linear Model Estimations: Tests of the Effect of Perceived Shared Condemnation, Within-Scenario Average Condemnation ($CONDEMNATION_k$), Within-Individual Average Condemnation ($Perception\ Bias_j$), and Other Control Variables on Empathy

Moral Disgust	Coefficients		Standard Error	$\|t\|$	*df*	*p*-value
Model 1						
π_0						
Intercept θ_0	1.82	***	0.397	4.58	3271	< .001
Perception Bias γ_{01}	0.02		0.099	0.24	234	.807
CONDEMNATION β_{02}	−0.24	**	0.080	2.98	28	.006
Perceived Shared Condemnation π_1						
Intercept θ_1	−0.23	***	0.015	15.42	3271	< .001
Model 2						
π_0						
Intercept θ_0	1.54	***	0.385	4.00	3269	< .001
Sex γ_{01}	0.80	***	0.086	9.27	234	< .001
University γ_{02}	0.34	***	0.088	3.82	234	< .001
Perception Bias γ_{03}	−0.04		0.094	0.37	234	.710
CONDEMNATION β_{04}	−0.24	**	0.080	3.02	28	.005
Perceived Shared Condemnation π_1						
Intercept θ_1	−0.12	***	0.020	6.27	3269	< .001
Sex γ_{11}	−0.18	***	0.023	7.87	3269	< .001
University γ_{12}	−0.08	***	0.023	3.46	3269	< .001

After confirming the main hypothesis regarding the association between perceived shared condemnation and moral emotions, we investigated whether each of the three moral emotions would predict willingness to punish the violator (‘fine’ and ‘wallet’). Again, since each participant responded to 15 norm violation scenarios (i.e. the willingness to punish scores and moral emotion scores were nested within each individual), the hierarchical linear modelling approach was employed. The models we tested are as follows:

Level 1 (within individuals)

$$Y_{ij} = \pi_{0i} + \pi_{1i}(\text{outrage}_{ij}) + \pi_{2i}(\text{disgust}_{j}) + \pi_{3i}(\text{empathy}_{j}) + \epsilon_{ij}$$

where Y_{ij} = individual i’s punitive intent (‘wallet’ or ‘fine’) for scenario *j*.

Level 2 (individual-level)

$$\pi_{0i} = \beta_{00} + \beta_{01}(\text{sex}_i) + \beta_{02}(\text{university}_i) + \beta_{03}(\text{set}_i) + \gamma_{0i}$$

$$\pi_{1i} = \beta_{10} + \beta_{11}(\text{sex}_i) + \beta_{12}(\text{university}_i) + \beta_{13}(\text{set}_i)$$

$$\pi_{2i} = \beta_{20} + \beta_{21}(\text{sex}_i) + \beta_{22}(\text{university}_i) + \beta_{23}(\text{set}_i)$$

$$\pi_{3i} = \beta_{30} + \beta_{31}(\text{sex}_i) + \beta_{32}(\text{university}_i) + \beta_{33}(\text{set}_i)$$

where sex was coded as 0 = female and 1 = male, and ‘set’ corresponds to Sets A vs. B.

The results of the series of hierarchical linear models are summarized in Tables S5 and S6. Moral outrage predicted willingness to impose a fine (see coefficient of β_{10} in Table S5) after controlling for potentially confounding variables. The effect of moral disgust on ‘fine’ was only marginally significant, and the effect of empathy was not significant (see coefficients of β_{20} and β_{30} in Table S5, respectively). For ‘wallet’, moral outrage, moral disgust and diminished empathy significantly predicted participants’ unwillingness to tell the norm violator about the dropped wallet (see coefficients of β_{10}, β_{20}, and β_{30} in Table S6).

Table S5 | Summary of Hierarchical Linear Model Estimations: Effects of Three Emotions (Moral Outrage, Moral Disgust, and Empathy) on Willingness to Impose a Fine

Impose a Fine	Coefficients		Standard Error	$\|t\|$	*df*	*p*-value
π_0						
Intercept β_{00}	0.24		0.200	1.19	232	.235
Sex β_{01}	−0.08		0.190	0.44	232	.663
University β_{02}	−0.20		0.206	0.99	232	.332
Scenario Set β_{03}	3.72	***	0.214	17.42	232	< .001
Moral Outage π_1						
Intercept β_{10}	0.98	***	0.054	18.11	3268	< .001
Sex β_{11}	0.02		0.048	0.36	3268	.716
University β_{12}	0.05		0.051	0.89	3268	.373
Scenario Set β_{13}	−0.71	***	0.057	12.43	3268	< .001
Moral Disgust π_2						
Intercept β_{20}	0.08	+	0.042	1.84	3268	.067
Sex β_{21}	−0.00		0.049	0.00	3268	.997
University β_{22}	−0.06		0.052	1.13	3268	.259
Scenario Set β_{23}	−0.49	***	0.051	9.52	3268	< .001
Empathy π_3						
Intercept β_{30}	0.04		0.127	0.34	3268	.737
Sex β_{31}	0.11		0.142	0.76	3268	.448
University β_{32}	−0.08		0.157	0.54	3268	.590
Scenario Set β_{33}	−1.24	***	0.143	8.66	3268	< .001

Table S6 | Summary of Hierarchical Linear Model Estimations: Effects of Three Emotions (Moral Outrage, Moral Disgust, and Empathy) on Unwillingness to Point out the Dropped Wallet

Wallet	Coefficients		Standard Error	|*t*|	*df*	*p*-value
π_0						
Intercept β_{00}	1.58	***	0.148	10.68	232	< .001
Sex β_{01}	0.08		0.183	0.44	232	.657
University β_{02}	−0.04		0.195	0.21	232	.834
Scenario Set β_{03}	0.57	***	0.169	3.40	232	< .001
Moral Outage π_1						
Intercept β_{10}	0.24	***	0.041	5.74	3268	< .001
Sex β_{11}	0.08	$^{+}$	0.044	1.76	3268	.079
University β_{12}	−0.12	**	0.045	2.72	3268	.007
Scenario Set β_{13}	−0.13	**	0.043	3.08	3268	.002
Moral Disgust π_2						
Intercept β_{20}	0.14	***	0.033	4.30	3268	< .001
Sex β_{21}	−0.04		0.044	0.90	3268	.366
University β_{22}	0.08	$^{+}$	0.046	1.78	3268	.075
Scenario Set β_{23}	−0.13	***	0.037	3.45	3268	< .001
Empathy π_3						
Intercept β_{30}	−0.22	*	0.096	2.24	3268	.025
Sex β_{31}	0.02		0.113	0.18	3268	.858
University β_{32}	−0.18		0.124	1.44	3268	.149
Scenario Set β_{33}	−0.31	**	0.108	2.89	3268	.004

Study 2

Method

Participants and Design. Participants were 102 undergraduate students at Kobe University, Japan (40 males and 62 females; mean age ± s.d. = 18.96 ± 0.70 years old). The study took approximately one hour to complete, and was conducted in a small group setting, comprising 2 to 12 participants. Participants were paid 1,000 Japanese yen (JPY) in exchange for their participation (1,000 JPY ≈ 8 Euros).

In each session, participants were required to read six norm violation scenarios, which were adopted from Study 1. Three of the six scenarios were associated with medium levels of moral outrage in Study 1 (mean ± s.d. = 2.34 ± 1.43, 2.60 ± 1.50, and 2.75 ± 1.53 for Scenarios 1, 12, and 23, respectively), and the other three were associated with low levels of moral outrage (mean ± *SD* = 0.85 ± 1.00, 1.12 ± 1.16, and 1.18 ± 1.11 for Scenarios 17, 19, and 21, respectively). We excluded the scenarios that had elicited intense moral outrage in Study 1 to avoid the ceiling effect. Of the six scenarios, two (one medium outrage scenario and one low outrage scenario) were accompanied by information indicating high levels of shared condemnation. Another two scenarios (one medium outrage scenario and one low outrage scenario) were accompanied by information indicating low levels of shared condemnation. The remaining two scenarios were fillers, and were not accompanied by any information. Participants were assigned to either the HL (high-low) or LH (low-high) condition. In the HL condition, the first two scenarios were accompanied by high shared condemnation information, and the last two scenarios were accompanied by low shared condemnation information. In the LH condition, the first two scenarios were accompanied by low shared condemnation information, while the last two were accompanied by high shared condemnation information. The middle two scenarios

were always filler scenarios that were not accompanied by any information. The filler scenarios were included to mitigate possible carryover effects of the previously presented information.

The effect of scenario order was mitigated by a semi-Latin square design. The six scenarios were ordered as Scenarios 1, 19, 12, 17, 23, and 21 by alternating medium and low emotion scenarios. Therefore, there were three pairs of medium and low outrage scenarios (1-19, 12-17, and 23-21). The Latin square design applied to these pairs yielded three orders. Participants were randomly assigned to one of the three order conditions.

In sum, Study 2 technically employed a 2 (information order: HL vs. LH) × 3 (scenario order) between-participants design. However, the primary concern of Study 2 was the effect of information (high vs. low levels of shared condemnation). Therefore, we analysed the data focusing on the effect of this within-participant factor.

Information of Shared Condemnation (Independent Variable). The level of sharedness of condemnation was manipulated by presenting hypothetical opinion distributions associated with the norm violation scenarios. Participants were told that a similar experiment had been conducted in the previous school-term, and they would be exposed to the results of that experiment. Specifically, for each of the four non-filler scenarios, participants were presented a bar chart purportedly depicting the distribution of previous participants' responses to the badness item: *How bad do you think the norm violation is?* (1 = 'not bad at all' to 4 = 'extremely bad'). To avoid presenting unrealistic distributions, we created four bar charts based on the actual distributions observed in Study 1, although the scenarios were not correspondent with the scenarios used in Study 2. The bar charts presented to participants are shown in Figure S1: Figures S1a and S1b correspond to the high shared condemnation condition, and Figures S1c and S1d correspond to the low shared condemnation condition. In the high shared condemnation

condition, the bar charts indicated that modal response was 4 (extremely bad). In the low shared condemnation condition, the modal response was 2 (not terribly bad). After presenting the information of the previous participants' opinions, the experimenter explicitly told participants that the information was provided just for their reference, and they should not conform to the previous participants' opinions.

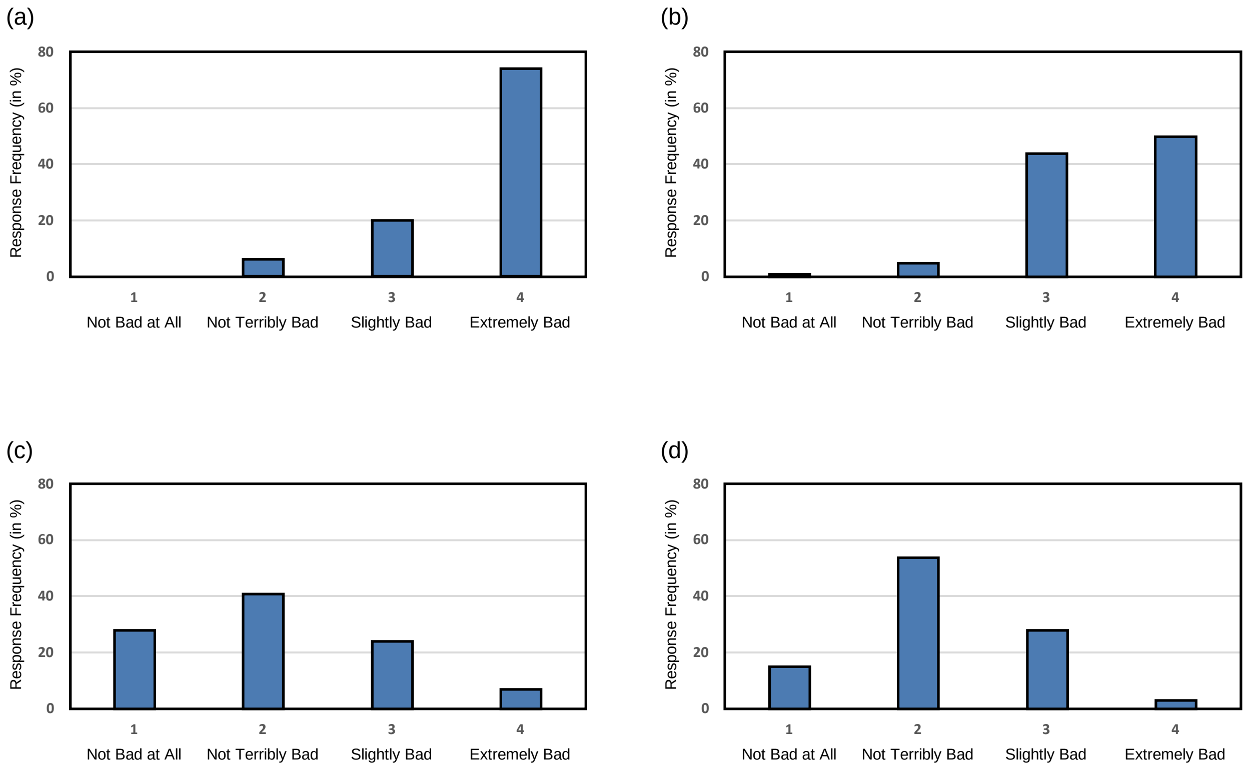

Fig. S1 | The opinion distributions presented to participants in Study 2. (a, b) The opinion distributions indicating that most previous participants considered the violation 'extremely bad'. (c, d) The opinion distributions indicating that most participants considered the violation 'not terribly bad'.

Procedure. The experimenter explained the nature of the experiment and then asked participants to sign an informed consent form. The experimenter distributed a booklet containing six norm violation scenarios. Each participant received one of three booklets corresponding to one of the three scenario orders. The experimenter then asked participants to read the first scenario. After confirming that all participants had finished reading it, the experimenter showed one of the two distributions (Fig. S1a in the HL condition or Fig. S1c in the LH condition). Participants then reported their moral emotions, perceived condemnation, perceived illegality, and willingness to punish the violator. These measures were the same as those used in Study 1. Participants then read the second scenario, were exposed to one of the two distributions (Fig. S1b in the HL condition or Fig. S1d in the LH condition), and then responded to the same items. For the middle two scenarios, participants read the scenarios and immediately responded to the items. For the last two scenarios, the procedures were the same as with the first two scenarios, except that participants were exposed to different opinion distributions.

After this study, participants took part in an unrelated study. At the end of the experimental session, participants were fully debriefed and paid 1,000 JPY. The data of Study 2 were analysed using the ezANOVA package of R (https://www.r-project.org/).

Results

Manipulation Check. The perceived shared condemnation item (*What proportion of Japanese citizens do you think would condemn this violator?*) was included to perform a manipulation check in Study 2. It was expected that participants would estimate higher levels of shared condemnation after receiving the high shared condemnation information (Figs. S1a and S1b) than after receiving the low shared condemnation information (Figs. S1c and S1d). Perceived condemnation was submitted to a 2 (information: high vs. low shared condemnation) ×

2 (scenario outrage: medium vs. low) analysis of variance (ANOVA) with both independent variables as within-participant factors. The main effects of information and scenario were significant, $F_{1, 101} = 65.25$, $p < .001$, $\eta_G^2 = .18$ for information, and $F_{1, 101} = 102.48$, $p < .001$, $\eta_G^2 = .24$ for scenario. The interaction between information and scenario was not significant, $F_{1, 101} = 2.79$, $p = .098$. As can be seen in Fig. 2a in the main text, participants believed that Japanese citizens would condemn the medium outrage violations more than the low outrage violations. More importantly, they expected that a greater proportion of Japanese citizens would condemn the violations accompanied by the high shared condemnation information than those accompanied by the low shared condemnation information.

Hypothesis Testing. After confirming that the manipulation was successful, we then tested the primary hypothesis that perceived shared condemnation would amplify moral outrage and moral disgust, and diminish empathy for the violator. As explained in the main text, the three moral emotion scores were submitted to a series of 2 (information: high vs. low shared condemnation) ×2 (scenario outrage: medium vs. low) ANOVA with both factors as within-participant factors. Although we conducted a series of ANOVAs including other control variables—three scenario order, information order [HL vs. LH], and sex—these control variables were not associated with main effects, except for the main effect of scenario order on empathy. Accordingly, we decided to report the analyses with a simpler design. The results are graphically summarized in Figs. 2b, 2c, and 2d in the main text for moral outrage, moral disgust, and empathy, respectively (for test statistics, see Table S7).

The two punitive intent variables (‘fine’ and ‘wallet’) were submitted to comparable 2 (information: high vs. low shared condemnation) × 2 (scenario outrage: medium vs. low) ANOVAs. The main effect of shared condemnation information was significant for the fine

version of punitive intent (see Figs. 2e and 2f in the main text, and see Table S8 for test statistics).

Table S7 | Summary of 2 (Scenario Outrage: Medium vs. Low) × 2 (Information: High vs. Low Shared Condemnation) ANOVAs with Moral Outrage, Moral Disgust, and Empathy as the Dependent Variables

	df_N	df_D	SS_N	SS_D	F		p	η_G^2
**Moral Outrage**								
Shared Condemnation Information (H vs. L)	1	101	7.20	107.16	6.79	*	.011	0.024
Scenario Outrage (Medium vs. Low)	1	101	147.60	109.42	136.24	***	1.94×10^{-20}	0.332
Interaction	1	101	0.29	80.56	0.36		.551	0.001
**Moral Disgust**								
Shared Condemnation Information (H vs. L)	1	101	1.73	204.22	0.86		.357	0.004
Scenario Outrage (Medium vs. Low)	1	101	1.73	114.57	1.53		.219	0.004
Interaction	1	101	0.44	134.55	0.33		.566	0.001
**Empathy**								
Shared Condemnation Information (H vs. L)	1	101	0.09	31.79	0.28		.598	0.001
Scenario Outrage (Medium vs. Low)	1	101	26.71	48.97	55.10	***	3.70×10^{-11}	0.193
Interaction	1	101	0.09	30.75	0.29		.592	0.001

Notes. Subscripts N and D designate ‘numerator’ and ‘denominator’, respectively. In the right-most column, η_G^2 designates generalized η^2.

Table S8 | Summary of 2 (Scenario Outrage: Medium vs. Low) × 2 (Information: High vs. Low Shared Condemnation) ANOVAs with Punitive Intent ('Fine' and 'Wallet')

	df_N	df_D	SS_N	SS_D	F		p	η_G^2
**Fine**								
Shared Condemnation Information (H vs. L)	1	101	6.88	131.37	5.29	*	.023	0.021
Scenario Outrage (Medium vs. Low)	1	101	9.73	99.52	9.87	**	.002	0.029
Interaction	1	101	0.12	91.13	0.13		.716	0.000
**Wallet**								
Shared Condemnation Information (H vs. L)	1	101	0.98	67.52	1.47		.229	0.006
Scenario Outrage (Medium vs. Low)	1	101	0.08	54.41	0.16		.687	0.001
Interaction	1	101	0.01	49.49	0.02		.888	0.000

Notes. Subscripts N and D designate 'numerator' and 'denominator', respectively. In the right-most column, η_G^2 designates generalized η^2.

Study 3

Method and Materials

Respondents. Respondents were recruited through an online survey service provided by a Japanese online research company, Cross Marketing Inc. Although we did not ask their ethnicity, it can be safely assumed that most respondents were Japanese because (i) they were currently living in Japan, and (ii) completed the survey in Japanese. A total of 834 respondents (421 males and 413 females; mean age ± s.d. = 44.17 ± 14.51 years old) completed the survey. However, as 147 individuals did not follow the instructions, responses from 687 respondents were retained in the subsequent data analyses.

Survey. As explained in the main text, the survey consisted of five sections and an additional screening section: (i) description of a norm violation in an open-ended format, (ii) victim(s), (iii) norm violator(s), (iv) perceived shared condemnation and indirect victimization, and (v) intervention.

In the screening section, demographic information (e.g., sex, age) was collected. In the first section, respondents described a personal experience of witnessing someone commit a moral or norm violation. To investigate third-party emotional reactions, respondents were explicitly told to report an incident in which they themselves had not been directly involved. In addition, they were told that a violation that they had directly observed would be more desirable than a violation that they had learned about via mass media or the Internet. The excluded 147 respondents either failed to describe a witnessed violation or did not follow the above instructions (e.g., reporting an incident in which they themselves had been harmed).

In the second section, respondents first indicated whether there had been an individual victim, a collective victim or no victim involved in the incident. When the incident involved an

individual victim, they reported the nature of their relationship with the victim (e.g., family, romantic partner, friend, stranger) and sense of closeness to the victim. When the incident involved either an individual or collective victim, participants reported their emotional reactions to the victim. The emotional reaction items were the same as those used in Studies 1 and 2 to measure emotional reactions to the norm violator (i.e., moral outrage, moral disgust, and empathy). The primary interest was empathy, as we did not expect respondents to have experienced negative emotions, such as outrage and disgust, in relation to the victim.

In the third section, respondents indicated whether the norm violator was an individual or a group. If the norm violator was an individual, they reported the nature of their relationship with the violator and sense of closeness to the violator. Respondents then reported their emotional reactions (moral outrage, moral disgust, empathy) to the norm violator.

The fourth section assessed perceived shared condemnation. Respondents first reported whether they had witnessed others condemning the violation. The response categories were as follows: ‘many people were condemning it’, ‘a couple of people were condemning it’, ‘one person was condemning it’, and ‘did not witness someone condemning it’. This variable is henceforth referred to as *witnessed condemnation*. Then respondents estimated what proportion of Japanese citizens would condemn it, and what proportion of their intimate friends would condemn it. The responses to these items (estimated percentages) were highly correlated with each other, $r_{685} = .72$, $p = 2.2\times10^{-16}$. Therefore, we averaged these two responses as the *perceived shared condemnation* score. In addition, respondents rated whether the violation had indirectly damaged them on a 5-point scale (1 = ‘not at all’ to 5 = ‘damaged a lot’).

In the fifth section, respondents reported whether they had intervened in the violation in some way. If they answered ‘yes’, they were asked to briefly describe how they had intervened.

They were further asked to what extent they had been driven by the following three motivations on a five-point scale (1 = 'not at all' to 5 = 'very much'): wanted to punish the violator, wanted to compensate the victim's damage, and wanted to restore justice/fairness.

Results

The characteristics of the reported violations are summarized in Table S9, and Table S10 shows the descriptive statistics and correlation matrix of the variables of interest. As can be seen in Table S10, witnessed condemnation was significantly correlated with perceived shared condemnation (r = .26). However, perceived shared condemnation was more strongly correlated with moral emotions. Therefore, in the subsequent analyses we focused on perceived shared condemnation.

We conducted a series of multiple regression analyses to examine whether perceived shared condemnation would predict each of the three moral emotions after controlling for the presence of a victim/victims and degree of indirect damage to self, along with respondents' sex and age. The presence of a victim/victims was represented by two dummy coded variables: (0, 0), (1, 0), and (0, 1) for 'no victim', 'individual victim', and 'collective victim', respectively. As shown in Table S11, the presence of an individual victim increased moral outrage and moral disgust. Unexpectedly, it also increased empathy for the violator. Involvement of indirect personal damage significantly increased moral outrage and moral disgust, whereas its effect on empathy was not significant. Even after controlling for these potentially confounding variables, perceived shared condemnation was positively associated with moral outrage and moral disgust, and negatively with empathy.

Table S9 | Characteristics of the Reported Violations

	(1) Freq.	(2) Rel. Freq.	(3) Rel. Freq. Within
The Presence of a Victim/Victims			
Individual Victim	218	.317	--
Collective Victim	139	.202	--
No Victim	330	.480	--
Relationship with Individual Victim (If Present)			
Family/Relative	6	.009	.028
Romantic Partner	1	.001	.005
Friend	29	.042	.133
Acquaintance	26	.038	.119
Stranger	144	.210	.661
Other	12	.017	.055
Presence of Individual Violator			
Yes: Individual Violator	568	.827	--
No: Collective Violator	119	.173	--
Relationship with Individual Violator (If Present)			
Family/Relative	8	.012	.014
Romantic Partner	6	.009	.011
Friend	30	.044	.053
Acquaintance	53	.077	.093
Stranger	446	.649	.785
Other	25	.036	.044
Witnessed Anyone Condemning the Violation			
Witnessed Many People Were Condemning It	142	.207	--
Witnessed a Few People Were Condemning It	143	.208	--
Witnessed One Person Was Condemning It	47	.068	--
Did Not Witness Someone Condemning It	355	.517	--
Did You Somehow Intervene?			
Yes	126	.183	--
No	561	.817	--

Notes. (1) ‘Freq.’ column includes frequencies of the corresponding responses, (2) ‘Rel. Freq.’ column includes relative frequencies of the responses in the entire sample, and (3) ‘Rel. Freq. Within’ column includes relative frequencies of the responses within the relevant sub-samples.

Table S10 | Descriptive Statistics and Correlation Matrix of the Variables of Interest with Cronbach's α Coefficients of the Three Moral Emotions in the Diagonal Cells (*N* = 687 for all cells)

		Mean (*SD*)	1	2	3	4	5	6
1	Witnessed Condemnation	2.90 (1.24)	- -					
2	Perceived Shared Condemnation	67.69 (30.34)	**.26*****	- -				
3	Moral Outrage	4.27 (1.27)	**.15*****	**.27*****	(.92)			
4	Moral Disgust	3.56 (1.47)	**.24*****	**.25*****	**.71*****	(.90)		
5	Empathy for Violator	1.74 (1.05)	.08*	−.09*	−.11**	**.12****	(.88)	
6	Indirect Damage to Self	2.74 (1.27)	.01	.04	**.25*****	**.20*****	.07	- -
7	Intervention	0.18 (0.39)	.05	.06	**.18*****	**.14*****	−.09*	**.22*****

Note. Correlation coefficients emphasized by bold font are significant after correcting the *P*-values by Holm's method.

Table S11 | Results of a Series of Multiple Regression Analyses Predicting the Three Moral Emotions from Perceived Shared Condemnation, Respondents' Sex and Age, the Presence of a Victim/Victims, and Degree of Indirect Damage to Self

	β		SE	|*t*|	*p*
Moral Outrage					
Perceived Shared Condemnation	.226	***	.036	6.27	6.53×10^{-10}
Sex (female = 0, male = 1)	−.046		.036	1.29	.198
Age	.055		.035	1.56	.120
Individual Victim	.155	***	.038	4.05	5.67×10^{-5}
Collective Victim	.056		.038	1.46	.145
Indirect Damage to Self	.255	***	.036	7.07	3.80×10^{-12}
Moral Disgust					
Perceived Shared Condemnation	.218	***	.036	5.99	3.35×10^{-9}
Sex (female = 0, male = 1)	−.004		.036	0.11	.913
Age	−.024		.036	0.66	.508
Individual Victim	.198	***	.039	5.12	4.03×10^{-7}
Collective Victim	.026		.039	0.68	.495
Indirect Damage to Self	.210	***	.036	5.76	1.31×10^{-8}
Empathy					
Perceived Shared Condemnation	−.099	*	.038	2.57	.010
Sex (female = 0, male = 1)	.111	**	.038	2.92	.004
Age	−.059		.038	1.56	.118
Individual Victim	.099	*	.041	2.41	.016
Collective Victim	.020		.041	0.49	.626
Indirect Damage to Self	.073	$^{+}$	.038	1.91	.057

According to Batson et al.[46], apparent moral outrage can be subsumed to empathic anger, which is trigged by harm to a cared-for other, but not by the norm violation per se. To test this possibility, we analysed a subset of the dataset: 218 violations involving an individual victim. We conducted a series of multiple regression analyses to test whether the effect of perceived shared condemnation on moral emotions would remain significant after controlling for empathy for the victim. The results are summarized in Table S12. The effect of perceived shared

condemnation on moral outrage and moral disgust remained significant even after controlling for empathy for the victim.

Table S12 | Results of a Series of Multiple Regression Analysis Predicting the Three Moral Emotions from Perceived Shared Condemnation, Respondents' Sex and Age, Empathy for the Victim, and Degree of Indirect Damage to self (Only Violations Involving an Individual Victim)

	β		SE	|*t*|	*p*
Moral Outrage					
Perceived Shared Condemnation	.191	**	.067	2.86	.005
Sex (female = 0, male = 1)	−.116	+	.066	1.76	.081
Age	−.003		.066	0.05	.958
Empathy for Victim	.194	**	.066	2.96	.003
Indirect Damage to Self	.179	**	.066	2.71	.007
Moral Disgust					
Perceived Shared Condemnation	.149	*	.067	2.21	.028
Sex (female = 0, male = 1)	−.083		.067	1.25	.214
Age	−.121	+	.067	1.82	.071
Empathy for Victim	.181	**	.066	2.72	.007
Indirect Damage to Self	.167	*	.067	2.49	.014
Empathy for Violator					
Perceived Shared Condemnation	−.076		.057	1.33	.186
Sex (female = 0, male = 1)	.064		.056	1.13	.259
Age	−.031		.056	0.55	.585
Empathy for Victim	.544	***	.056	9.73	2.00×10^{-16}
Indirect Damage to Self	.144	*	.056	2.55	.012

Studies 1 and 2 revealed that perceived shared condemnation modulated the intensity of moral emotions when no readily identifiable victim was present. To confirm this observed pattern, we analysed the sub-sample of the dataset: 330 norm violations where the respondents reported no obvious victim had been involved. The results are summarized in Table S13. Confirming the results of Studies 1 and 2, when no obvious victim was involved, perceived shared condemnation was positively associated with moral outrage and moral disgust, whereas the effect of perceived shared condemnation on empathy became only marginally significant.

Table S13 | Results of a Series of Multiple Regression Analyses Predicting the Three Moral Emotions from Perceived Shared Condemnation, Respondents' Sex and Age, and Degree of Indirect Damage to Self (Only Violations without an Obvious Victim)

	β		SE	|*t*|	*p*
Moral Outrage					
Perceived Shared Condemnation	.236	***	.051	4.63	5.34×10^{-6}
Sex (female = 0, male = 1)	.039		.051	0.78	.439
Age	.062		.051	1.23	.221
Indirect Damage to Self	.300	***	.051	5.88	1.02×10^{-8}
Moral Disgust					
Perceived Shared Condemnation	.244	***	.052	4.66	4.59×10^{-6}
Sex (female = 0, male = 1)	.082		.052	1.58	.114
Age	.008		.052	0.15	.880
Indirect Damage to Self	.223	***	.052	4.26	2.66×10^{-5}
Empathy					
Perceived Shared Condemnation	−.108	$^{+}$	.055	1.96	.051
Sex (female = 0, male = 1)	.126	*	.054	2.32	.021
Age	−.126	*	.055	2.32	.021
Indirect Damage to Self	.021		.055	0.38	.706

We then tested whether moral emotions would predict intervention after controlling for potentially confounding variables (i.e., respondents' sex and age, presence of an individual victim, presence of a collective victim, degree of indirect damage to self). As shown in Table S10, moral outrage and moral disgust were extremely highly correlated (r = .77). Therefore, to avoid the multicollinearity problem, instead of entering all three moral emotions together in a single logistic regression model, we conducted two separate regression analyses. As shown in Table S14, both moral outrage and moral disgust predicted intervention (when two moral emotions were entered in the single model, neither outrage nor disgust was significant). The results indicate that those who felt an intense feeling of outrage and/or disgust were more likely to intervene in the violation.

However, closer scrutiny of intervention behaviours revealed that many of them did not qualify as costly third-party punishment (e.g. calling the police). To compare the relative importance of the three potential motivations for intervention, the three motivation scores (i.e. motivations to punish the violator, compensate the victim, and restore fairness/justice) were submitted to a 2 (sex) × 3 (motivation) ANOVA with the latter factor as repeated measures. Only the main effect of motivation was significant ($F_{2, 248} = 7.61$, $P < .001$, $\eta_p^2 = .058$). The motivation to restore fairness/justice (3.77 ± 1.15) was significantly higher than the motivation to punish the violator (3.32 ± 1.41; $t_{248} = 3.07$, $p = .002$) and the motivation to compensate the victim (3.24 ± 1.46; $t_{248} = 3.62$, $p < .001$).

Table S14 | Results of the Logistic Regression Analyses Predicting Intervention from Moral Emotions, Respondents' Sex and Age, Presence of a Victim/Victims, and Degree of Indirect Damage to Self

	β		SE	OR	|Z|	*p*
Constant	−1.76	***	.12	0.17	14.51	$2.00{\times}10^{-16}$
Moral Outrage	0.30	*	.12	1.35	2.43	.015
Empathy	−0.32	**	.12	0.72	2.66	.008
Sex (female = 0, male = 1)	0.13		.11	1.14	1.22	.221
Age	0.34	**	.11	1.40	3.15	.002
Individual Victim	0.42	***	.12	1.52	3.58	$3.51{\times}10^{-4}$
Collective Victim	0.23	*	.11	1.26	2.10	.036
Indirect Damage to Self	0.59	***	.11	1.80	5.12	$3.13{\times}10^{-7}$
Constant	−1.75	***	.12	0.17	14.56	$2.00{\times}10^{-16}$
Moral Disgust	0.23	*	.11	1.26	2.13	.033
Empathy	−0.39	**	.12	0.68	3.20	.001
Sex (female = 0, male = 1)	0.13		.11	1.14	1.22	.223
Age	0.35	***	.11	1.43	3.33	$8.77{\times}10^{-4}$
Individual Victim	0.42	***	.12	1.53	3.61	$3.02{\times}10^{-4}$
Collective Victim	0.25	*	.11	1.28	2.22	.026
Indirect Damage to Self	0.61	***	.11	1.84	5.39	$6.98{\times}10^{-8}$

Notes. The OR column indicates the odds ratios. The two models differ only in the shaded variables (i.e., moral outrage and moral disgust).

Although the punitive motivation was less important than the fairness/justice motivation, it is noteworthy that moral outrage and moral disgust were more strongly associated with the punitive motivation than the fairness/justice motivation (Table S15). These patterns suggest that individuals intervened in norm violations for various reasons (e.g. to restore fairness/justice), and that not all intervention behaviours were driven by emotional reactions. Rational considerations may facilitate more adequate interventions, such as calling the police, in modern societies. Such cool-headed interventions may not be driven by moral emotions; however, moral emotions seem to play some role and urge people to punish a violator for its own sake.

Table S15 | Correlations between the Three Moral Emotions (Outrage, Disgust, Empathy) and the Three Motivations of Intervention (Motivations to Punish the Violator, Compensate the Victim, and Restore Fairness/Justice)

	Mean	*SD*	Moral Outrage		Moral Disgust		Empathy
Punishment	3.32	1.41	**.29**	**	**.29**	**	−.14
Compensation	3.24	1.46	.07		−.07		−.10
Fairness/Justice	3.77	1.15	0.20	*	.17	+	.00

Note. Correlation coefficients emphasized by bold font are significant after correcting the *P*-values by Holm's method.
